# A Digital Intervention to Improve Skin Self-Examination Among Survivors of Melanoma: Protocol for a Type-1 Hybrid Effectiveness-Implementation Randomized Trial

**DOI:** 10.2196/52689

**Published:** 2024-02-12

**Authors:** Sharon Manne, Carolyn J Heckman, Sara Frederick, Alexis A Schaefer, Christina R Studts, Olga Khavjou, Amanda Honeycutt, Adam Berger, Hao Liu

**Affiliations:** 1 Rutgers Cancer Institute of New Jersey New Brunswick, NJ United States; 2 University of Colorado Anschutz Medical Campus Aurora, CO United States; 3 RTI International Research Triangle Park, NC United States

**Keywords:** melanoma, cancer survivorship, skin self-examinations, digital interventions

## Abstract

**Background:**

Although melanoma survival rates have improved in recent years, survivors remain at risk of recurrence, second primary cancers, and keratinocyte carcinomas. The National Comprehensive Cancer Network recommends skin examinations by a physician every 3 to 12 months. Regular thorough skin self-examinations (SSEs) are recommended for survivors of melanoma to promote the detection of earlier-stage, thinner melanomas, which are associated with improved survival and lower treatment costs. Despite their importance, less than a quarter of survivors of melanoma engage in SSEs.

**Objective:**

Previously, our team developed and evaluated a web-based, fully automated intervention called mySmartSkin (MSS) that successfully improved SSE among survivors of melanoma. Enhancements were proposed to improve engagement with and outcomes of MSS. The purpose of this paper is to describe the rationale and methodology for a type-1 hybrid effectiveness-implementation randomized trial evaluating the enhanced MSS versus control and exploring implementation outcomes and contextual factors.

**Methods:**

This study will recruit from state cancer registries and social media 300 individuals diagnosed with cutaneous malignant melanoma between 3 months and 5 years after surgery who are currently cancer free. Participants will be randomly assigned to either enhanced MSS or a noninteractive educational web page. Surveys will be collected from both arms at baseline and at 3, 6, 12, and 18 months to assess measures of intervention engagement, barriers, self-efficacy, habit, and SSE. The primary outcome is thorough SSE. The secondary outcomes are the diagnosis of new or recurrent melanomas and sun protection practices.

**Results:**

Multilevel modeling will be used to examine whether there are significant differences in survivor outcomes between MSS and the noninteractive web page over time. Mixed methods will evaluate reach, adoption, implementation (including costs), and potential for maintenance of MSS, as well as contextual factors relevant to those outcomes and future scale-up.

**Conclusions:**

This trial has the potential to improve outcomes in survivors of melanoma. If MSS is effective, the results could guide its implementation in oncology care and nonprofit organizations focused on skin cancers.

**International Registered Report Identifier (IRRID):**

RR1-10.2196/52689

## Introduction

### Background

More than 97,610 cases of cutaneous malignant melanomas will be diagnosed in the United States in 2023, making it the fifth most commonly diagnosed cancer. With incidence rates more than tripling from 1975 to 2020, the population of survivors of melanoma is estimated at >1 million. Among persons diagnosed with localized, regional, or distant-stage melanoma, the 5-year survival rates have improved in the last decade, with 99%, 65%, and 25% survival rates, respectively [1]. Improved survival and increased incidence have translated to an estimated annual treatment cost for melanoma of approximately US $2.5 billion [2].

Survivors of melanoma remain at risk of recurrence, second primary cancers, and keratinocyte carcinomas. Recurrence rates depend on tumor thickness and nodal involvement and range from 3% to 24% among persons with thinner lesions to 51% among persons with thicker lesions or lymph node involvement [3-5]. Recurrent or new primary melanomas occur most commonly during the first 5 years after diagnosis but can arise many years later [6,7]. Recurrent melanoma typically occurs at local or regional sites, and approximately half of distant recurrences present within the skin or lymph nodes [8,9]. Psychosocial morbidity is an issue for patients and survivors, with studies reporting higher anxiety and depressive symptoms than in the general population [10] and elevated anxiety about recurrence.

For survivors of melanoma, National Comprehensive Cancer Network guidelines recommend several actions, including skin examinations by a physician every 3 to 12 months and regular thorough skin self-examination (SSE) [11-13], which entails a deliberate, systematic inspection of all areas of the body using a mirror or the assistance of another person to examine hard-to-view areas [14]. Patient education about SSE by a health care professional is also recommended. Engagement in regular sun protection behavior is also recommended to reduce the risk of subsequent skin cancers as UV radiation from the sun is a contributing factor for melanoma and other skin cancers [2].

Limited data suggest that performance of SSE is associated with differential survival rates among patients with melanoma, and professional recommendations for regular SSE are supported by 3 research findings. First, more than half of recurrences and new primary melanomas are detected by survivors themselves [11,15,16]. Second, individuals who perform SSE are diagnosed with significantly earlier-stage melanomas than those who do not. Detection and treatment of recurrent disease and new primary cancers at earlier stages leads to improved survival, which is not accounted for by lead-time bias [17]. Third, melanomas identified through SSE are thinner than those found incidentally [16,18,19]. Retrospective studies suggest that individuals who perform SSE have lower tumor thickness. Thinner melanomas are associated with better survival [19-23]. Thus, promoting regular SSE will likely enhance the early detection of easier-to-treat recurrences and new primary cancers among survivors. For example, Robinson et al [24] found that 13.4% of patients in their SSE randomized controlled trial (RCT) developed a new melanoma over the 2-year follow-up. Patients in the SSE intervention condition detected 81% of these melanomas, approximately 34% of which were invasive, with only a 1% increase in physician visits. The costs of treating earlier- versus later-stage melanomas are significantly lower [25,26]. In summary, regular thorough SSE is recommended for survivors of melanoma and results in the detection of earlier-stage, thinner melanomas, which is associated with improved survival and lower treatment costs.

Despite its importance, engagement in regular, thorough SSE is low. Compliance rates with thorough SSE range between 7% and 17% among survivors of melanoma [14,20,22,27,28]. Studies document wide variation in SSE performance, with figures dependent upon the way that SSE is measured and the time frame for assessment. When patients are asked whether they have performed any form of SSE in the past 2 months, high rates of performance are observed (71.5%) [29]. SSE rates are higher when specifying any performance in the past year (84.3%) [30]. However, significantly lower rates are found if SSE is defined by its thoroughness. We reported that 13.7% of survivors checked 4 key areas and had someone assist them or used a mirror for hard-to-see areas [30]. Loescher et al [31] found that 16% of women and 7% of men examined each of the 7 designated body parts in the previous 2 months. Mujumdar et al [32] reported that 17% of survivors examined a minimum number of areas of the body (8 out of 9 areas) in the previous 2 months. In our recent work, 65% reported having conducted an SSE in the previous 2 months, but only 7.5% of the sample checked all 15 body parts. Hard-to-see areas were missed, including the scalp (37.9%), buttocks (40.4%), soles of the feet (41.6%), and genitals (44.4%). Less than half (46.2%) [33] reported using a mirror to view hard-to-see places, and only 39% reported asking for assistance. In total, 2% reported using a mole map to guide their most recent SSE.

A limited number of interventions have been evaluated to improve SSE among survivors of melanoma [24,34-37]. Existing interventions have used in-person, print, or web-based delivery modes. Several have demonstrated improvements in SSE [34,35]. Limitations of previous studies include lack of a comparison group [35]; being an in-person or partner-assisted intervention, which compromises the ability to disseminate it [24]; lack of inclusion of long-term outcomes [24]; lack of inclusion of SSE performance [36] as an outcome; and low intervention use and high dropout [35]. No existing intervention is fully automated and, thus, potentially cost-effective and highly scalable. To this end, our team developed and evaluated mySmartSkin (MSS), a web-based, fully automated intervention to improve SSE among survivors of melanoma. MSS is a behaviorally based program that is delivered via the internet, tailored to the user, and fully automated with no human clinical support. MSS was compared with usual care (UC) in an RCT of 430 survivors of melanoma from New Jersey [38,39]. The results indicated a beneficial impact of MSS versus UC on the performance of thorough SSE at all follow-ups up to 1 year [24,34-37]. Effect sizes were in the small to medium magnitude range for SSE, but these effects were not sufficiently strong to scale up for implementation in the existing form.

In addition to SSE, professional agencies recommend engagement in regular sun protection behaviors, such as staying in the shade, applying sunscreen with a sun protection factor of at least 30, and wearing protective clothing (eg, hats and long sleeves). Survivors of melanoma report engaging in higher levels of sun protection behaviors than the general population [40], but their sun protection behaviors do not meet the recommended guidelines [30,32,41]. To date, only 2 intervention studies have targeted improved sun protection behaviors among survivors of melanoma [34,38]. Bowen et al [34] reported significant improvements in some sun protection behaviors (eg, wearing sunglasses and staying in the shade). In our prior work [38], we evaluated the effects of MSS on sun protection behaviors, which was included as a component of the original intervention. The effects of MSS compared with UC on sun protection behaviors were only statistically significant at the 24-week follow-up in analyses that did not control for baseline sun protection behaviors or other potential covariates. Stronger and more consistent improvements in sun protection would be an important goal for future work.

### Theoretical Frameworks

Previous empirical findings regarding factors associated with SSE [29,30,32,42,43] and sun protection, as well as the preventive health model (PHM) [44,45], inform the content of MSS. The PHM posits that the performance of preventive behaviors is influenced by background, affective, cognitive, and social factors. Background factors include sociodemographic characteristics, risk factors for skin cancer, medical history, and knowledge about melanoma. Affective factors include concerns about melanoma recurrence and distress about the diagnosis. Cognitive factors include perceived controllability of melanoma, self-efficacy, and benefits and barriers. Social normative factors include family and friend support and physician recommendations. In addition to our previous research on skin cancer risk reduction practices among family members of patients with melanoma [46,47], the PHM has been used successfully to understand and promote screening for colorectal and prostate cancer [44,48]. The original MSS focused on improving the perceived controllability of melanoma through the performance of comprehensive SSE; increasing the perceived benefits of SSE and sun protection; reducing barriers to SSE and sun protection; enhancing self-efficacy for SSE and sun protection; and enhancing family, friend, and physician support for sun protection.

**Figure 1:**
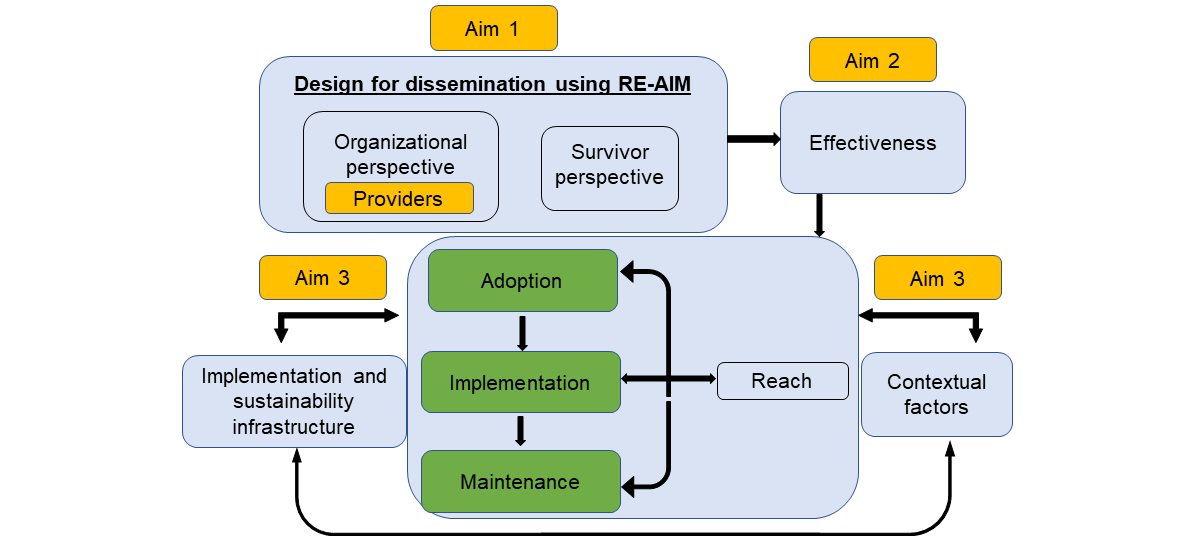
Integration of preventive health model and implementation frameworks with the study aims. RE-AIM: Reach, Effectiveness, Adoption, Implementation, and Maintenance.

A major challenge for intervention research is that most interventions that demonstrate a beneficial impact are not tested in effectiveness or dissemination trials and are not conducive to “real world” delivery or use. As a result, they do not penetrate the general population and, unfortunately, are not received by those who most need them [49]. Our work is guided by the Practical, Robust Implementation and Sustainability Model (PRISM), which extends the Reach, Effectiveness, Adoption, Implementation, and Maintenance (RE-AIM) [50] framework to consider not only implementation outcomes but also multilevel contextual factors influencing implementation. *Reach* refers to the percentage and representativeness of persons exposed to a program. *Effectiveness* refers to the impact on key survivor-level outcomes. As MSS is a free-standing, recipient-facing intervention, *adoption* is defined as the proportion and representativeness of recipients (in this case, survivors) who adopt (ie, at least log into) the program. *Implementation* refers to the degree to which a program is delivered and received as intended. This is measured through costs, engagement, acceptability, feasibility, and appropriateness. *Maintenance* is the extent to which a program and its survivor-level effects are sustained over time. The integration of these models with the study’s aims is shown in Figure 1 [51-55]. In the proposed study, in aim 1, the goal of the iterative process of enhancing the MSS intervention using stakeholder feedback and usability testing is to improve RE-AIM outcomes, which will be assessed in aims 2 and 3. In aim 2, we focus on the effectiveness of the enhanced MSS, testing its effects on survivor-level outcomes, including clinical outcomes (eg, melanomas found). In aim 3, we address the remaining RE-AIM dimensions. In addition, to proactively identify barriers to and facilitators of future scale-up and widespread dissemination and implementation of MSS, we explore multilevel contextual factors identified by key stakeholders drawn from the PRISM domains of recipients, external environment, intervention design, and implementation and sustainability infrastructure. We anticipate that incorporating PRISM and RE-AIM throughout the study aims will ensure that the enhanced intervention is responsive to key stakeholder preferences and that we “design for dissemination” [56], recognizing potential barriers to and facilitators of future scale-up and informing our next stage of developing dissemination and implementation strategies to maximize the public health impact of MSS.

### Study Objectives

#### Overview

The purpose of this type-1 hybrid effectiveness-implementation study is to evaluate the effectiveness of a web-based intervention, MSS, and explore its implementation and relevant contextual factors. A type-1 hybrid effectiveness-implementation approach requires the engagement of multilevel stakeholders throughout the research process, focus on evaluating the effectiveness of the enhanced MSS, and identification of critical factors that could promote or prevent wide-scale implementation. The study’s 3 aims are guided by implementation frameworks and behavior change theories.

#### Aim 1

The first aim is to enhance MSS by collaborating with multilevel stakeholders. We will collaborate with stakeholders to enhance MSS through qualitative interviews and usability testing of potential intervention enhancements. Enhancements are based on empirically validated behavior change techniques (BCTs) and lessons learned from survivors’ feedback in our previous study. We will use an iterative process that includes (1) key informant interviews with survivors, providers, and professional organization representatives regarding proposed enhancements; (2) conversion to an enhanced mobile-based delivery platform; (3) usability testing; and (4) iterative program refinements.

#### Aim 2

The second aim is to evaluate the effects of the enhanced MSS on thorough SSE (primary outcome) and examine its impact on the diagnosis of new or recurrent melanomas (secondary outcome) and sun protection practices (secondary outcome). We will conduct an RCT comparing MSS and a noninteractive educational web page with 300 survivors to test the intervention effects on these outcomes.

#### Aim 2 Hypothesis

We propose that MSS participants will be more likely to perform thorough SSE over the 18-month follow-up period. We will explore the impact of MSS on new or recurrent melanomas and sun protection behaviors. We anticipate diagnosing a greater number of earlier-stage melanomas and observing higher sun protection behaviors among those in MSS compared with those in UC.

#### Aim 3

The third aim is to assess selected implementation outcomes and identify factors relevant to future scale-up for widespread dissemination, implementation, and maintenance of MSS. We will use mixed methods to assess implementation outcomes and explore perspectives from survivors, care providers, and professional organizations to inform the selection of strategies to best disseminate and implement MSS on a broad scale. The subaims are as follows:

1. Aim 3a is to estimate program costs and assess the cost-effectiveness of MSS relative to control. We hypothesize that MSS costs will be higher than UC costs. We expect that MSS will be a more cost-effective strategy given its previous positive effects on SSE and the identification of new or recurrent melanoma. If our findings support this as expected, exploratory cost-effectiveness analyses from the health care and societal perspectives will be conducted using simulation models of melanoma-related costs, disease progression, and survival over 5- and 10-year analysis horizons.
2. Aim 3b is to examine the reach, adoption, implementation (ie, engagement, acceptability, appropriateness, and feasibility), and maintenance of MSS. For reach, we predict that the demographic characteristics of those exposed to MSS will not differ from those of the general population of survivors of melanoma. For adoption, we propose that the proportion of contacted and eligible survivors randomized to MSS who consent, complete the baseline questionnaire, and log into MSS will be equal to or greater than the proportion in our previous efficacy trial. For engagement, we propose that 80% of MSS participants will log into the intervention at least once. For acceptability, we predict that MSS will be rated as highly acceptable. We do not have hypotheses regarding appropriateness, feasibility, and maintenance as these data will be evaluated in terms of future implementation of MSS should it have an impact on SSE and sun protection outcomes. For each of these implementation outcomes, we will assess the demographic characteristics of MSS participants to determine whether outcomes differ among survivors in specific subgroups (eg, by age, sex, race, ethnicity, and socioeconomic status).
3. Aim 3c is to identify and describe contextual factors experienced by multilevel stakeholders as key to scale-up and widespread implementation of MSS, including consideration of potential delivery settings, timing of delivery, and resources needed to promote its implementation.

## Methods

### Ethics Approval

Study procedures were approved by the Rutgers University institutional review board (IRB; protocol Pro2022000948).

### Aim 1: Enhancements to the Existing Version of MSS Using Multilevel Stakeholders

#### Overview

This phase engages multilevel stakeholders in optimizing MSS by targeting multiple RE-AIM outcomes: effectiveness, adoption, implementation, and maintenance. Iterative enhancements will be made to MSS intended to increase the effects on SSE by increasing adoption, acceptability, appropriateness, feasibility, and maintenance. This process will include key informant interviews with survivors, health care delivery stakeholders, and professional organizations to inform the selection of enhancements from the potential theory-based strategies we identified based on the findings and survivor feedback from our previous RCT. This user-centered process will involve assessment of preferences regarding content (eg, “What are your thoughts on the option of uploading pictures of your moles?”), use (eg, “How useful would it be to log in to MSS three months after you complete it and what would you use it for?”), and preferred ways to motivate targeted behaviors (eg, “What are your thoughts about incentives such as free products?”). This phase of our design for dissemination approach will use an iterative process that includes (1) key informant interviews with survivors of melanoma and care providers to inform the selection of proposed theory-based enhancements, (2) conversion to a mobile-based intervention delivery platform incorporating selected enhancements, (3) usability testing with survivors who use the program for 1 month, and (4) program refinement.

#### Proposed Enhancements

The existing MSS content was guided by PHM and evidence-based BCTs. The original MSS included two categories of BCTs: (1) prompts (participants could set an email reminder to perform SSE on specific dates for the duration of study participation) and (2) planning (participants could set a goal related to SSE and select up to 2 action steps to address barriers). The other material primarily targeted SSE knowledge (eg, how to recognize a suspicious growth) and self-efficacy (building confidence in identifying suspicious growths), which were the mediators of MSS effects on SSE.

Our selection of proposed enhancements was guided by two goals: (1) to optimize engagement with MSS and (2) to increase sustained performance of SSE. A summary of enhancements is shown in Textbox 1. We focused on optimizing engagement as participants who used MSS more were more likely to perform SSE across the follow-up period, which we are now extending. Thus, increasing engagement with MSS should enhance its impact over time.

**Proposed enhancements to mySmartSkin (MSS).**

**Self-monitoring and performance feedback**

- Upload pictures of moles each month on mole map
- Illustrate body parts checked each month
- Bar graph of completed skin self-examinations (SSEs) over time from baseline to 1 year on the MSS home page
- Monthly feedback on progress with SSE

**Incentives for success**

- Incentives for self-reported SSE, core completion, and SSE and sun protection goal setting: rulers, reminder stickers, sun hats, sunscreen, sun protection factor ChapStick, sun safety checklist magnet, calendars, hanging door signs with the ABCDEFs (asymmetry, border irregularity, color variation, diameter of >6 mm, evolving, and funny looking) of melanoma, sun umbrella, among others (up to 6 incentives)

**Prompts**

- Personalized text reminder delivered the day before SSE is due each month to perform it, with a link to the self-check program
- Tailored reminder of body parts to remember to check

**Goals and planning**

- Identification of personal barriers to SSE and sun protection behaviors
- Continued troubleshooting of ways to address SSE barriers and sun protection behaviors
- Regular personalized feedback and ability to track progress on SSE and sun protection goals

**Social support**

- Discuss the importance of support for change
- Identify someone to support SSE (eg, spouse) and how the patient would like to talk to and involve them
- Provide SSE information via email to authorized support person

To optimize initial engagement, we are implementing four enhancements: (1) enhancing participant-facing recruitment materials (eg, advertisements, emails, and study home page) by including narratives from survivors and oncologists about MSS and the importance of thorough SSE, (2) streamlining enrollment materials and processes (eg, screening and baseline surveys and instructions), (3) reaching out to participants who do not log into MSS by day 3 to provide a brief orientation session fostering ease of access (ie, review modules and topics and the importance of completing modules, show the participant how to log in, and discuss SSE benefits for finding recurrence early), and (4) informing participants about incentives for completion of each core and other tasks within the program.

To improve sustained SSE, we identified 6 categories of BCTs for the proposed enhancements [57]; we included (1) self-monitoring and performance feedback to improve self-efficacy (eg, uploading photos of moles and saving them to compare with the next SSE); (2) behavioral incentives (eg, provided for completing SSE); (3) additional prompts for SSE (eg, programmed reminders about missed areas of the body); (4) adding a more comprehensive approach to setting goals and planning, including continued troubleshooting of barriers; (5) including more content on receiving assistance and support from others in performing SSE (including the option to email an authorized support person about assisting with the participant’s SSE); and (6) adding a goal-setting component that fosters habit formation and maintenance of behavior change. We created an in-depth goal-setting section that allows users to track their progress and make updates to their goals and progress over time. The goal-setting component is tailored based on the user’s current SSE and sun protection behaviors, and the purpose of this component is to assist in the creation of a plan to improve both behaviors.

In terms of sun protection behaviors, we included a sun safety core with content categorized into chapters addressing (1) learning more about sun safety with topics such as risks of UV rays, risks of unprotected sun exposure, sunscreen education, how to avoid sunburns, and other sun protection behaviors; (2) assessing current sun protection behaviors; (3) increasing confidence in and motivation for sun protection; and (4) setting sun protection goals. A more detailed summary of the content of each core is provided in Table 1.

**Table 1:** Enhanced mySmartSkin intervention content.

Section	Key content	Interactive features
Tutorial	Overview of navigation, sections, and features	N/A^a^
Core 1: learn about spots	Personal melanoma history Skin cancer facts and figures Melanoma risk factors Risk of recurrence Purpose of SSE^b^ How to conduct an SSE The ABCDEFs^c^ of melanoma Your experience doing SSE What to do when you find a suspicious spot Importance of SSE and physician skin examinations Confidence in checking spots	Mole facts and fiction ABCDEF-identifying challenge quiz Suspicious or not mole challenge Vignette and physician video and audio clips SSE goal setting
Core 2: sun-safe behaviors	Sun-safe behaviors Risks of sun exposure UV index Types of sunscreen Avoiding sunburns during outdoor activities Risk assessment for outdoor activities Sun-safe clothing Limiting sun exposure Increasing confidence and motivation for sun safety Prioritizing sun-safe behaviors SSE goal setting	Sunscreen FAQs^d^ Sunscreen facts and fiction Tanning facts and fiction Assessing current tanning beliefs Vignette and physician video or audio clips Sun-safe behavior importance rating Sun safety goal setting
MyStuff	Goal summary SSE progress tracker Badges and prizes	Goal progress tracker

^a^N/A: not applicable.

^b^SSE: skin self-examination.

^c^ABCDEF: asymmetry, border irregularity, color variation, diameter of >6 mm, evolving, and funny looking.

^d^FAQ: frequently asked question.

#### Key Informant Interviews to Gather Input on Enhancements

This phase of aim 1 has been completed. The procedures are described in the following sections, and the results are described later in the paper (see the *Results* section).

##### Sample

We planned to recruit 10 survivors of melanoma who had completed treatment within up to 3 years since diagnosis from the Rutgers Cancer Institute of New Jersey (CINJ) through new case ascertainment by research staff using electronic medical records. On the basis of our past experience in qualitative assessments, this sample was expected to provide thematic saturation of the data, although additional interviews could be conducted if needed.

##### Questionnaire

Following informed consent procedures, participants completed a brief demographic questionnaire before their interview began. Participants self-reported their age, sex or gender, race, ethnicity, education, current insurance coverage, school enrollment, employment, and marital status. Patients also reported current SSE practices.

##### Procedures

The project coordinator (AS) conducted the interviews, which were audio recorded with participant permission and approximately 45 minutes in duration. During the interviews, participants were shown prototypes and wireframe illustrations of the proposed enhancements. To ensure that feedback was gathered on all aspects of the MSS platform, interviewers highlighted different sections, including the goal-setting activity sequence and skin self-check body map. The semistructured interviews elicited feedback on the proposed enhancements. We included probes about enhancements based on stakeholders’ positive, neutral, or negative reactions (eg, “What parts of the site did you think were most interesting and helpful?” and “What parts were less helpful or seemed less relevant to you?”). Phase 1 participants received US $50 for the interviews. These interviews were conducted in person at the CINJ or using videoconferencing software. The interviewer took field notes, and the interviews were transcribed verbatim.

##### Refining Enhancements

The study team reviewed the field notes and verbatim transcriptions of key informant interviews. The responses to each proposed enhancement were coded as positive, neutral, negative, or mixed. Although potential enhancements were based on findings from the previous RCT, suggestions from participants, BCTs, and proven ways to optimize engagement, if particular enhancements were perceived negatively by multiple stakeholders, we used these interviews to explore alternatives that would be more acceptable. For those enhancements with multiple negative or mixed comments, the team decided whether the issue could be addressed feasibly and crafted changes to the content and approach that were congruent with stakeholder feedback. The stakeholders in later interviews reviewed the changes made in response to feedback given in earlier interviews to provide confirmation on whether the issue was adequately addressed. The study team provided feedback to the web developer, Radiant (Radiant Creative Group LLC), throughout the interview process for changes to be incorporated in a timely manner.

#### Conversion to a Mobile-Based MSS Platform, Usability Testing, and Refinements With Multilevel Stakeholders

Stakeholder-informed enhancements to content and program features will be incorporated with the existing content and migrated to the mobile-based delivery platform in collaboration with Radiant. Testing will be conducted throughout the preproduction and production stages to ensure the suitability of the content for the intended audience. Weekly meetings with Radiant will be conducted to review uploaded content.

#### Usability Testing, Feedback, and Refinements

Testing will be accomplished by conducting focused interviews with up to 10 survivors of melanoma, 5 health care providers, and 5 organization representatives who will each receive US $100. Testing is modeled after a National Cancer Institute website design project [58]. The process will involve checking the content with participants for attractiveness, comprehension, acceptability, and persuasion. In addition, participants will be able to use MSS for approximately 1 week before the interview so that we can gather input on features that are planned over time, such as self-monitoring and performance feedback, incentives, and prompts. A series of questions and structured guides will be prepared in advance related to the elements to be evaluated.

In the set of interviews, all enhancements and engagement tools, including reminder prompts, will be activated, providing an opportunity to evaluate all components of the enhanced MSS. After that, we will set up a virtual meeting in which the participant will provide feedback about acceptability, ease of use or intuitiveness, and satisfaction. During this session, we will discuss the topic areas and review MSS using a shared screen to allow participants to react to each screen. Participants will be asked to provide feedback on features. These sessions will be digitally video recorded and transcribed, and AS will take notes. The data will be summarized and reviewed to determine further modifications to be made. These efforts will yield a set of understandable, acceptable, and appealing enhancements to MSS that are customized to the needs, capabilities, and preferences of patients with melanoma, providers, and organizational stakeholders. We will conduct weekly meetings that include Radiant and the study team to review and produce content. Once the final beta website has been agreed upon by the full research team, it will undergo extensive testing by Radiant to ensure that the programming is functioning correctly.

### Aim 2: Evaluate Effects of MSS Versus a Noninteractive Educational Web Page on SSE

#### Overview

Phase 2 is a randomized effectiveness trial in which MSS will be tested in an RCT comprising 2 groups (MSS vs a noninteractive educational web page) and 5 assessments (baseline, 3 months, 6 months, 1 year, and 18 months) with 300 survivors of melanoma. The main goal is to evaluate the effects of MSS on thorough SSE and examine the impact on clinical outcomes (eg, diagnosed early-stage skin cancers).

#### Recruitment

##### Overview

We will recruit participants from 2 sources: state cancer registries and Facebook. This approach was adopted to prepare for dissemination by assisting the team in comparing the reach of the 2 methods of recruitment. This will foster our ability to determine future settings. The registries include all patients with melanoma in each state, which facilitates eventual generalizability. We chose NJ and CA registries because the 2 states are geographically disparate and sociodemographically diverse, and we have worked successfully with both registries on current projects (CA2219854 and CA221854). We chose Facebook as a second approach as the vast majority of Americans (84% in 2021) [59] use social media, with use steadily increasing each year. This strategy will reach a broad audience of survivors. In addition, many melanoma and skin cancer advocacy groups are active on social media, which will inform the potential for working with them on future dissemination.

##### Registries

We will recruit from the New Jersey State Cancer Registry (NJSCR) and the Cancer Registry of Greater California (CRGC). Owing to state laws, allowable recruitment procedures differ. NJSCR procedures entail case ascertainment, a letter to the physician, an address check, a letter mailed to the patient, up to 12 calls to verify that they received the letter, and the provision of verbal consent to send contact information to the CINJ. The CINJ reaches out to the patient to describe the study and send a link to the MSS website. We have enhanced the CRGC procedures to be as similar as possible to those of the NJSCR. The CRGC queries their database, confirms that the patient is alive, and checks this information with the provider. Case information is sent to the CINJ, a letter is sent explaining the study followed by up to 12 calls to the patient to obtain their email address, and a link to the study web page is emailed. The process takes approximately 2 months.

##### Facebook

We will use paid Facebook advertisements. We will work with a social media marketing company to develop keywords and advertisements targeting individuals with characteristics similar to those of survivors of melanoma (eg, older age). As noted previously, this approach has been successfully used in 2 previous and one current skin cancer–related intervention project [60-62]. This approach will also recruit a broader population of survivors of melanoma than the previous efficacy trial.

##### Eligibility

The eligibility for this trial is as follows:

1. Diagnosis of primary pathological stage-0 to stage-III cutaneous malignant melanoma
2. Being 3 months to 5 years after surgery
3. No current evidence of cancer
4. No adherence to thorough SSE [14] (ie, did not check the entire body at least once during the past 3 months)
5. Age of ≥18 years
6. Internet access
7. Ability to speak and read English
8. Ability to provide informed consent

The incidence is most common among non-Hispanic White individuals, who have an annual rate of 28 cases per 100,000 compared with 7 in American Indian or Alaska Native individuals; 5 in Hispanic individuals; and 1 in non-Hispanic Black, Asian, and Pacific Islander individuals (92.4% among non-Hispanic White individuals). Consistent with population rates, we will oversample minority groups from cancer registries to ensure a minimum of 7.6% racial and ethnic minority group survivor enrollment.

#### Sample

The projected sample size is 300. Participants will be recruited using 2 methods. Half of the sample will be recruited from 2 state cancer registries. We expect >10,000 eligible patients with melanoma from the 2 registries during our recruitment period. We will select a random sample from each diagnosis year to balance the year of diagnosis. The acceptance rate for the previous MSS study was 40.9%, but we anticipate a lower recruitment from the registry participants in this study based on our recent work with both CRGC and NJSCR patients (CA221854) [63]. Both cancer registries have a large number of survivors annually. Thus, we will randomly select a subset of cases from each of the retrospective 5-year time frames. The other half of the sample will be recruited via Facebook. We will use paid Facebook advertisements targeted at persons who follow skin cancer–related causes and organizations**.** We will end recruitment from each source when the required sample size is reached.

#### Enrollment, Randomization, Intervention Delivery, and Follow-Up Surveys

Potential participants recruited via registries will be asked for their email when they verbally consent to participate in the study. Staff will then email these potential participants the link to the study’s web page. Participants recruited via social media advertisements will be automatically linked to the study web page when they click on the “learn more” part of the advertisement. Once on the web page, the following automated, completely web-based procedures will be used for enrollment. On the study’s web page, there is a screening survey to determine eligibility. Potential participants will complete the eligibility screener. If eligible, the program automatically moves to the electronic consent. Once consent is obtained, the program automatically moves to the baseline survey. After survey completion, the program randomizes the participant to MSS or the comparison condition and automatically moves to either the MSS landing page or the comparison condition landing page. Randomization will be stratified according to disease stage and time since diagnosis (3 months-3 years and 4-5 years) and, then, within strata, block randomized using blocks of 20 participants overseen by the study statistician following standard operating procedures of the CINJ Biostatistics Shared Resource.

Participants who do not log into the MSS program by day 3 will be contacted by a study coordinator via SMS text message, email, or phone 3 times between days 3 and 10 to remind them about logging into MSS. Participants who log in but do not review core 1 by day 5 will be contacted 3 times via email, SMS text message, or calls to invite them to review core 1. These interactions will also serve as an orientation for the participants allowing them to ask any questions about the study or the intervention.

#### Survey Procedures

Participants will be prompted (via automated email, as well as via SMS text message, telephone, and mail, as necessary) to complete the following web-based surveys: follow-up 1 at 3 months after the baseline, follow-up 2 at 6 months after the baseline, follow-up 3 at 1 year after the baseline, and follow-up 4 at 18 months after the baseline. Participants who do not complete the surveys will be reminded as follows: (1) reminder email and SMS text message with a link to the survey if it is not returned after 1 week, (2) call with a voicemail if no contact is made and email with a link to the survey if it is not returned after 2 weeks, and (3) the same procedure as at 2 weeks if the survey is not returned after 3 weeks. Participants who do not return the survey after a month will be considered lost to follow-up at that time point. Our primary effectiveness analyses will focus on individuals’ performance of thorough SSE at 18 months, which will ensure that all participants’ SSE practices are assessed over a long time frame and increase the likelihood of detecting new or recurrent melanomas. Participants will receive incremental gift card amounts for each survey (maximum total of US $120).

#### Intervention Procedures

Participants will have a unique identifying user code and create a password to access the interventions. If users forget their password, they will provide a unique answer to a personal question and the database will return the forgotten password. If users forget both their user ID and password, they can contact the technical assistance hotline. In the previous study, navigation issues were rare. We anticipate that it will take participants approximately 1 hour to complete the entire MSS intervention once and approximately 15 minutes to complete the comparison condition. Participants will have access to the interventions for 18 months.

#### Comparison Group

We chose a noninteractive educational web page comparison group for the following reasons: (1) the information incorporated is widely available to the public and used in dermatology and primary care, (2) the digital delivery method is similar to that of MSS, (3) it allows for a comparison of MSS effects with a minimal knowledge-based educational intervention, (4) it permits a comparison of MSS effects with the results of previous studies that used minimal comparison interventions, (5) it presents a standardized intervention rather than a variable treatment-as-usual intervention, (6) a no-intervention control would be unethical for this high-risk population, and (7) we considered using an intervention for another health issue (eg, nutrition) but anticipated that survivors of melanoma would be less likely to participate. We will create a static educational web page whose content will be based on freely available, accurate information relevant to melanoma, melanoma risk, and SSE. Possible topics include the warning signs of melanoma [64], the risk of recurrence among survivors of melanoma, and how to perform an SSE [65].

#### Aim 2 Measures

##### Covariates

###### Demographics

The demographic information collected will include age, sex, race, ethnicity, education, marital status, state of residence, employment, income, and insurance.

###### Clinical Characteristics

The clinical characteristics collected will be date of diagnosis, stage, and treatment received.

###### Melanoma Risk

The melanoma risk information collected will be eye color, natural hair and skin color, skin reactivity to the sun, freckling, moles, sunburn history, indoor UV tanning, and family history.

###### Environmental Factors

The environmental factors collected will be month of assessment, residential location, and average UV index at solar noon over the 3 months before each time point.

##### Primary Outcome Measure: Skin Self-Check Comprehensiveness

A full list of outcome measures and covariates is shown in Table 2. The primary outcome consists of the following items: (1) “Have you, or anyone else, not including a doctor or other healthcare professional, ever checked any part of your body for signs of skin cancer?” (yes or no), (2) “If yes, in the last three months, did you check your body for early signs of skin cancer?” (yes or no), and (3) “Doing a thorough skin check or skin self-examination means spending time looking at the skin systematically and deliberately. Please indicate whether you thoroughly checked each of the following areas of your body the last time you checked your body for early signs of skin cancer: Scalp, face, neck, shoulders, front of arms, back of arms, chest, stomach, upper back, lower back, front of legs, back of legs, bottom of feet, buttocks, and genitals” (yes or no for each). For all time points, comprehensiveness will be based on the total number of body parts checked among participants who conducted an SSE in the last 3 months. Participants who did not conduct an SSE in the last 3 months will be coded as 0 for this outcome.

**Table 2:** Aim 2 measures.

Measure			Baseline		Time 2		Time 3		Time 4		Time 5
**Covariates**											
	Date of birth	✓									
	Sex	✓									
	Marital status	✓									
	Race and ethnicity	✓									
	Education	✓									
	Insurance status	✓									
	Employment status	✓									
	Time since diagnosis	✓									
	Month of assessment	✓		✓		✓		✓		✓	
	Residential location										
	Average UV index at solar noon	✓		✓		✓		✓		✓	
**Outcomes**											
	Skin self-examination	✓		✓		✓		✓		✓	
	Sun protection behaviors	✓		✓		✓		✓		✓	
	Melanoma diagnosis, stage, thickness, and date			✓		✓		✓		✓	
	Clinician confirmation of cancer			✓		✓		✓		✓	

##### Secondary Outcomes

###### Sun Protection Behaviors

Participants report how often they engage in 4 behaviors when outside on a sunny day: wearing sunscreen with a sun protection factor of ≥30, wearing a long-sleeved shirt, wearing a wide-brimmed hat, and staying in the shade [66]. Items are rated on a 5-point Likert scale (1=*never*; 5=*always*).

###### Clinical Outcomes

Participants report whether a new melanoma or other skin cancer was diagnosed and the stage, thickness, and date. Physician confirmation of this information will be collected.

##### Analyses of the Impact of MSS on SSE, Sun Protection, and Clinical Outcomes

###### Analyses of Thorough SSE and Sun Protection

Demographics and baseline variables will be compared between the 2 conditions using chi-square tests and ANOVAs for categorical and continuous variables, respectively. These variables, including outcomes, will also be compared by recruitment source. Missing data will be handled using multiple imputation with 50 imputed samples [67]. Tests of intervention effects on thorough SSE and sun protection will be conducted separately for each wave of postintervention data collection using logistic regression models that treat baseline SSE or sun protection, respectively, as a covariate. Given the large number of potential additional demographic and medical covariates, additional analyses will include the covariates that are significant predictors of the outcome.

###### Sample Size and Power

On the basis of our previous study, we expect follow-up survey completion rates between 80% and 93% at the 1-year time point and 80% to 85% at the 18-month time point, which will result in an expected final sample size of N=255. In the previous trial, thorough SSE occurred at a rate of approximately 30% in MSS at each time point, and effect sizes for conducting a thorough SSE ranged from *w*=0.29 (8 weeks) to *w*=0.21 (6 months). Given that the MSS intervention will be enhanced, we expect that the SSE rate will be higher (45%), which will result in approximately *w*=0.36. A sensitivity power analysis using G*Power indicated that a sample of N=255 will be able to detect an even smaller effect of *w*=0.22 with a 2-tailed test, Cronbach α of .05, and power of 0.95. Thus, the expected effect of *w*=0.36 with a sample size of N=255 will be detectable with a power of 0.99. Given that sun protection behavior is a secondary outcome, we are not powering the trial for this variable.

###### Analyses of the Impact of MSS on the Diagnosis of New or Recurrent Melanoma

The impact of MSS would be strengthened with data suggesting that survivors who use MSS detect suspicious growths and bring them to physicians for appropriate workup and diagnosis. Given the likely low recurrence rate over the 18 months that participants are followed for in this trial, the study is not powered for testing hypotheses on this end point. Rather, we will conduct an exploratory analysis to examine whether diagnoses differ between MSS and UC. Specifically, the number of diagnoses of earlier-stage new and recurrent melanoma at 18 months will be compared for the 2 conditions using either negative binomial regression models or binary logistic models depending on the distribution of new diagnoses (ie, if there are patients with more than one new diagnosis during the period). If our findings show that more melanomas are detected in MSS, we will conduct exploratory cost-effectiveness analyses from the health care and societal perspectives by creating simulation models of melanoma-related costs, disease progression, and survival over 5- and 10-year analysis horizons from the start of the trial (aim 3a).

#### Aim 3: Implementation Outcomes and Contextual Factors Relevant to Future Scale-Up

##### Overview

The type-1 hybrid effectiveness-implementation approach allows us to obtain vital data to inform future scale-up, dissemination, and implementation. This aim has 3 parts. In aim 3a, we will assess the costs of delivery of MSS and the control condition website; if indicated by the results of aim 2, we will conduct cost-effectiveness analyses. In aim 3b, guided by RE-AIM, we will evaluate the implementation outcomes of MSS from the aim 2 effectiveness trial. In aim 3c, we will elicit actionable feedback on contextual factors relevant to future scale-up, dissemination, implementation, and maintenance from multilevel stakeholders, including survivors, providers, and representatives of organizations invested in melanoma survivorship care. Quantitative and qualitative data from aims 3a, 3b, and 3c will be analyzed and interpreted together to provide valuable insights and directions regarding implementation strategies needed to inform the scale-up and widespread implementation, focusing particularly on reach, adoption, implementation, and maintenance. The results of aim 3 as a whole will answer the following key questions: how can we increase the number of MSS users to maximize adoption? What dissemination channels are preferred to increase our reach to survivors? How can MSS be maintained to provide a sustainable intervention for survivors? What resources or adaptations are needed for organizations and practices to integrate MSS into their survivorship care offerings?

#### Aim 3a: Costs and Cost-Effectiveness

##### Assessment of Program Costs

The research team will track the costs of delivering both MSS and the noninteractive educational web page. Costs will first be estimated from a program perspective, capturing the explicit resources required to deliver and maintain the program after all start-up costs (eg, development and programming costs) have been incurred. Costs that are needed to deliver the intervention will be collected to inform the cost of scale-up, but any research-related costs that would not be required for standard program implementation will also be tracked. Sunk costs, which are one-time upfront costs, will be reported separately from ongoing program implementation costs. Program cost data will be collected using a modified version of previous cost surveys developed by the research team and adapted for several interventions [68-73]. The cost surveys capture all relevant labor- and non–labor-related inputs necessary to quantify costs. Labor costs for program staff time (via questionnaires) will be valued using actual or estimated wages. Nonlabor costs will be obtained from program billing records; these include materials and supplies used to support program and intervention activities and costs of facilities and contracted services. The feasibility of including the value of participant time spent on the intervention will also be explored, where participants’ time will be valued using age- and sex-specific wage rates. In addition, if the results of the analysis in aim 2 indicate that more melanomas are found among MSS participants than among the educational web page arm participants, costs to the health care sector will be estimated. These include the costs of diagnosing and treating recurrent melanoma. Published estimates of stage-specific medical costs of melanoma will be used to calculate the treatment costs of melanoma by stage at detection [74-79]. To estimate costs from a societal perspective, the costs to each stakeholder group will be summed, including program implementation costs, costs to the health care sector, and the value of participants’ time.

##### Cost Analysis

Total and incremental program costs will be estimated for each arm. Program development costs, a one-time capital investment, will be calculated separately from ongoing implementation costs, or the costs to maintain program delivery for the duration of the intervention. Analyses will distinguish between fixed costs, those that do not vary with enrollment rates (eg, server maintenance), and variable costs, which increase for each participant added. As most costs for MSS are expected to be fixed, the mean cost per participant will be driven largely by the number of enrolled participants. To explore the costs of scale-up, sensitivity analyses will be conducted regarding additional program dissemination efforts and variations in uptake rates. The primary cost-effectiveness analysis will evaluate the additional costs per thorough SSE completed for MSS versus educational web page participants over the 18-month trial. This analysis will be conducted from the program, health care sector (including program costs and costs of health care use for lesions detected), and societal (ie, health care sector plus participant costs) perspectives. The incremental cost-effectiveness of MSS relative to the educational web page will be determined as the difference in mean costs between the 2 arms divided by the difference in mean outcome changes [80,81].

##### Simulation Models of Melanoma-Related Costs, Disease Progression, and Survival

If the findings show that more melanomas are detected in early stages for the MSS arm relative to the educational web page arm as expected, secondary or exploratory cost-effectiveness analyses will be conducted from the health care sector and societal perspectives by creating statistical simulation models of melanoma-related costs and disease progression over 5- and 10-year analysis horizons from the start of the trial. Assuming the equivalent likelihood of a melanoma recurrence for MSS and educational web page participants, trial data on stage at diagnosis for melanomas detected during the trial (from aim 2) and published estimates of average stage at diagnosis for survivor recurrences in the general population will be used. Melanoma treatment costs beyond the trial period will be simulated for both arms, where the hypothesis is that MSS participants will have lower treatment costs than those in the educational web page arm as earlier detection requires less aggressive treatment. Mortality will also be simulated for both arms to create an effectiveness measure of simulated life years gained (LYGs) for MSS versus the educational web page comparison group. To estimate LYGs, reductions in melanoma mortality for MSS versus the comparison group will be combined with life expectancy data [82]. As health-related quality of life among survivors of melanoma is similar to health-related quality of life in the general population, there will be no quality adjustment of LYGs as any differences across arms in quality-adjusted life years would be driven by differences in mortality [83-88]. The cost-effectiveness analysis will examine the total incremental costs of MSS compared with the educational web page arm. Incremental costs include program implementation costs (excluding sunk and research costs), health care use costs incurred during the trial and any melanoma treatment costs simulated to occur beyond the trial, and the value of patients’ time to participate in the intervention. Incremental effectiveness will be calculated as LYGs for MSS compared with the educational web page arm. Base analyses will discount future costs and life years and use bootstrapping methods to generate 95% CIs for the incremental cost-effectiveness ratio. Sensitivity analyses will explore factors such as rates of detection of thin lesions through SSE, which may make MSS more cost-effective than the educational web page comparator.

#### Aims 3b and 3c: Exploration of Implementation Outcomes and Contextual Factors Relevant for Future Scale-Up

##### Sample

###### MSS Participants

All participants in the intervention trial assigned to MSS will complete surveys assessing specific implementation outcomes. A subset of MSS participants (n=30) will be purposively selected to participate in key informant interviews to reflect a diversity of MSS outcomes, MSS use, ratings of acceptability, and sociodemographic and clinical characteristics.

###### Additional Stakeholders

To ensure that we obtain a breadth of perspectives on the potential for scale-up, dissemination, widespread implementation, and maintenance of MSS, we will also identify and invite 20 individuals from multiple groups involved in melanoma treatment and survivorship to participate in key informant interviews. These stakeholders will include additional health care providers and representatives from the same organizations participating in aim 1. Participants in aim 3 key informant interviews will receive US $50 for completing the interviews, which will be conducted in person or via videoconferencing software.

#### Procedures, Measures, and Analyses

##### Overview

Aim 2 addresses the effectiveness of MSS. Aim 3a addresses the costs associated with delivering MSS, an important factor in the future potential scale-up of the intervention. In aims 3b and 3c, we use mixed methods to focus on the remaining RE-AIM outcomes and PRISM domains using data from the aim 2 RCT combined with surveys and stakeholder interviews.

##### Quantitative Implementation Outcomes and Analyses

As this is a type-1 hybrid effectiveness-implementation study, our analyses of implementation outcomes are primarily descriptive and meant to inform future planning for scale-up, dissemination, and implementation. Analyses of *Reach* will describe the proportion of contacted individuals who express interest by completing an eligibility survey. The clinical and sociodemographic characteristics of potential participants will be described and compared with those of the population of survivors of melanoma. Analysis of *Adoption* will describe the proportion of eligible participants who enter the intervention website (Table 3). Analyses of *Implementation* will describe the indicators listed in Table 3 and examine their variability by participant clinical characteristics, sociodemographic characteristics, and melanoma risk factors assessed in aim 2. We will also examine the variability in indicators of implementation by recruitment source (registry vs social media advertisements). The Acceptability of Intervention Measure (AIM) is a brief, quantitative measure of intervention acceptability that has demonstrated content and structural validity and test-retest reliability [89] as well as face validity [90]. Participants respond to 4 statements using 5-point Likert-type scales, with higher scores indicating higher acceptability. The AIM will be completed by MSS arm participants at the first follow-up. Acceptability ratings will be summarized using descriptive statistics and compared across clinical, sociodemographic, and risk factor groups.

**Table 3:** Aim 3 implementation measures.

Outcome	Definition	Measurement	Data source
Reach	Proportion and representativeness of individuals who express interest in MSS^a^	Proportion of contacted participants who complete an eligibility survey Sociodemographic and medical characteristics of individuals who screen vs the population of survivors	Recruitment and enrollment data National survivor data set (eg, NHIS^b^ and HINTS^c^)
Adoption	Proportion of individuals who begin an intervention	Proportion of contacted participants who consent, complete baseline survey, and enter the intervention website	MSS automated data
Engagement	How much individuals use MSS	Number of core modules completed SSEs^d^ performed with self-check program Number of views of MSS Use of each enhancement (incentives)	MSS automated data
Program costs and cost-effectiveness	Total and incremental costs of MSS vs comparison arm; cost-effectiveness of MSS	Surveys and billing and invoicing documents	Staff, service provider, and participant surveys; billing and invoicing, including payments; and published literature
Feasibility	Perception that MSS is feasible for use, dissemination, and delivery	Qualitative key informant interviews	Health care providers and professional organizations
Acceptability	Perception that MSS is satisfactory	Acceptability of Intervention Measure Qualitative key informant interviews	Participant surveys Survivors, providers, and organizations
Appropriateness	Perception that MSS is an appropriate fit for intended use	Qualitative key informant interviews	Survivors, health care providers, and organizations
Maintenance	Perceptions of the likelihood, needs, and resources for maintaining MSS delivery	Qualitative key informant interviews	Health care providers and organizations
PRISM^e^ contextual factors	Perceived contextual factors related to scale-up and D&I^f^	Qualitative key informant interviews	Survivors, health care providers, and organizations

^a^MSS: mySmartSkin.

^b^NHIS: National Health Interview Survey.

^c^HINTS: Health Information National Trends Survey.

^d^SSE: skin self-examination.

^e^PRISM: Practical, Robust Implementation and Sustainability Model.

^f^D&I: dissemination and implementation.

##### Qualitative Key Informant Interviews

Interviews will be conducted with stakeholders from each group (ie, participants, providers, and organizations) to assess the perceptions of potential barriers and facilitators to consider for future scale-up, widespread implementation, and maintenance. MSS participant interviews will be conducted within the month following their final follow-up during the aim 2 trial. Interviews with other stakeholders will be conducted following completion of the effectiveness trial during the final study year. Semistructured interview guides will include open-ended questions and probes regarding [91] implementation outcomes of feasibility, acceptability, and appropriateness of widespread implementation of MSS for survivors of melanoma. The potential for MSS maintenance will also be explored using open-ended questions and probes, including resources available and needed for the sustainability of the intervention from multiple stakeholder perspectives. In addition, we plan to focus on three PRISM domains: (1) organizational, provider, and patient perspectives on the intervention; (2) characteristics of the recipients of the intervention (survivors); and (3) implementation and sustainability infrastructure (if MSS were to be disseminated and supported by specific organizations outside the research context). To elicit perspectives on the 3 PRISM domains of interest to scale-up, dissemination, implementation, and maintenance, interview questions (as appropriate for each stakeholder group) will focus on (1) current approaches and practices relevant to the promotion of SSE; (2) the benefits and shortcomings of the MSS intervention; (3) barriers to MSS use (for survivors and those who may suggest it as a resource); (4) possible strategies to disseminate MSS to survivors; (5) pros and cons of suggested dissemination channels and strategies; (6) the “fit” of MSS with the missions and objectives of providers, practices, and organizations; and (7) resources, infrastructure, and other factors needed to sustain MSS.

##### Qualitative Analyses of Implementation Outcomes

We will follow the guidelines established by the COREQ (Consolidated Criteria for Reporting Qualitative Research) for data collection, analysis, and reporting. The audio recordings of the interviews and focus groups will be transcribed verbatim, deidentified, and imported into the ATLAS.ti software (ATLAS.ti Scientific Software Development GmbH) for analysis. Directed content analysis will be used. This structured method allows researchers to specify constructs of interest a priori (eg, acceptability, feasibility, appropriateness, and PRISM domains) and obtain detailed descriptions or elaborations of them, classifying and coding themes within and across constructs [92]. The study investigators will develop a preliminary codebook for each stakeholder group. A primary and secondary coder will independently code and discuss an initial subset of interviews to explore thematic content and then merge and explore them for concordant and discordant coding. After refining the codebooks, they will recode the first set of transcripts and determine the interrater reliability. After achieving consistency, the coding process will continue. In total, 4 randomly selected transcripts will be identified for double coding and evaluation of discordant coding, followed by any needed modifications and recoding to achieve consensus. Once the initial coding has been completed, 10% of the sample (ie, 1-2 participants from each stakeholder group) will be randomly selected and invited to participate in a member-checking process to determine whether additional data collection is necessary and ensure that valid inferences are made through coding procedures [93]. Following any further corrections, the team will develop a summative grid of emergent themes across and within stakeholder groups.

##### Synthesis of Quantitative and Qualitative Data to Inform Scale-Up

Mixed methods data will be analyzed and integrated using a concurrent parallel design [94]. As described previously, each type of data (quantitative and qualitative) will initially be collected and analyzed separately. A mapping and matrix approach will facilitate the integration and interpretation of the results, and joint displays will present the integrated results, connecting qualitative themes with quantitative outcomes. The results will be summarized within and across stakeholder groups to allow for consideration of multilevel dissemination and implementation strategies needed for future scale-up, dissemination, implementation, and maintenance.

#### Data Safety and Monitoring

Before initiating this study, the Rutgers Scientific Review Board will review the study procedures to ensure their scientific merit, safety, legality, and technical feasibility per the established policy. The proposed procedures will then be reviewed for protection against risks by the Rutgers health sciences IRB. Adverse events will be reported through the Rutgers CINJ for processing as per the established policy. This policy includes specific timelines for reporting events that are stipulated by the IRB. In addition to the IRB, the Rutgers CINJ Protocol Monitoring Committee reviews all adverse events for investigator-initiated trials as they occur. The project coordinator, under the direct supervision of the principal investigator, will be responsible for reporting any adverse events that are documented on the safety or adverse events form or reported by the study interventionist. The Rutgers CINJ Protocol Monitoring Committee will oversee the validity and integrity of the data by conducting periodic audits of the study records. The committee is empowered to suspend or close studies with major deficiencies and provides direction to investigators in the development of corrective action plans to rectify and meet identified deficiencies. As part of the Protocol Monitoring Committee function, accrual is monitored for clinical trials. All clinical trials undergo a semiannual review by the Protocol Monitoring Committee, at which time accrual figures are reviewed. Specific accrual rates for each trial are required under protocol monitoring policy. The Rutgers CINJ uses an internal audit program to address retention of participants, adherence to protocol, and data completeness. This audit program is reviewed and governed by the Protocol Monitoring Committee. In addition, there is a Data Safety and Monitoring Advisory Board. This team will consist of a dermatologist, a psychologist, and a survivor of melanoma from the community. The board, along with the study principal investigators, will convene for an in-person or virtual meeting annually. The agenda of the annual meeting will be to review risk procedures, adverse event reporting, and quality assurance. The Data Safety and Monitoring Advisory Board will review any serious adverse events reported as well as investigator adherence to eligibility rules.

## Results

To date, we have completed the first phase of aim 1; the remaining phases of this aim are currently in progress. The prototype and content for the MSS intervention were developed in collaboration with Radiant Inc. Following the procedures described in the *Methods* section, one set of stakeholder interviews was conducted with 5 survivors of melanoma who were shown the SSE body map component. A second set of stakeholder interviews was conducted with another sample of 5 survivors of melanoma who were shown the goal-setting module. Participants were shown the prototypes and wireframe. The responses were summarized in field notes, and we reviewed the notes and verbatim transcriptions of key informant interviews. Each proposed enhancement was coded as supportive, neutral, negative, or mixed. For those enhancements that had multiple negative or mixed comments, the team decided whether the issue could be addressed and crafted changes to the content and approach that were congruent with stakeholder feedback.

For the 2 sets of interviews, 19 patients were approached. Of these 19 patients, 10 (53%) were enrolled and 9 (47%) refused. The sample consisted of 50% (5/10) women and 50% (5/10) men, ranging in age from 32 to 75 (mean 57, SD 14.55) years. They were primarily non-Hispanic White (9/10, 90%) individuals, with 10% (1/10) of the participants identifying as non-Hispanic Black. Most (9/10, 90%) were employed, and 50% (5/10) had a college degree or higher education. In total, 40% (4/10) had conducted a partial SSE in the last year, another 30% (3/10) had conducted a comprehensive SSE in the last year, and 30% (3/10) had not conducted an SSE in the last year. A summary of the comments regarding design and navigation suggestions is provided in Multimedia Appendix 1. A summary of comments about content is shown in Table 4.

The team provided the recommended changes based on the feedback to the web developer. The interviewees in later interviews reviewed the changes made in response to feedback given in earlier interviews to provide confirmation on whether the issue was adequately addressed.

**Table 4:** Feedback regarding mySmartSkin (MSS) content and coding for phase 1.

Feedback	Coding	Team decision
Mixed reviews on the incentive options	Mixed	Changed incentive options based on feedback
MSS would be very helpful for survivorship care	Positive	—^a^
Wish MSS was available for public use	Positive	—
Content is thorough but quick to get through, which will motivate patients to use the app	Positive	—
Helps user feel in control of their health	Positive	—
Content seems overwhelming	Mixed	As some users felt that the content was manageable whereas others felt that it was overwhelming, we added a time estimate for each chapter so users will know how long it will take to complete so they can break the content into manageable sessions based on their own schedule.
Make the app more fun	Negative	Included gamification aspects such as badges and prizes that users can win
The importance of SSE^b^ and MSS comes through in the content	Positive	—
Content is straightforward, direct, and concise	Positive	—
The app is visually pleasing	Positive	—
User likes the ability to make the text bigger	Positive	—
User likes the use of statistics to reinforce the importance of SSE	Positive	—
Want to share app with family and friends	Positive	—

^a^No decision made as feedback as positive.

^b^SSE: skin self-examination.

## Discussion

### Principal Findings

Once the analyses are completed, we anticipate that MSS participants will be more likely to perform thorough SSE and sun safety behaviors over the 18-month follow-up period and propose that there will be more earlier-stage melanomas diagnosed in MSS than in UC. In terms of the cost analysis, we expect that MSS will be a more cost-effective strategy given its greater effectiveness in increasing SSE and identifying new or recurrent melanoma. For the reach outcome, we predict that the demographic variables of survivors exposed to MSS will not differ from those of the general population of survivors of melanoma. For adoption, we propose that the proportion of contacted or eligible survivors randomized to MSS who consent, complete the baseline survey, and log into MSS will be equal to or greater than the proportion who adopted the intervention in our previous efficacy trial. For engagement, we propose that 80% of MSS participants will log into the intervention site at least once. For acceptability, we predict that MSS will be rated as highly acceptable, with mean acceptability ratings of ≥4 (out of 5) on the AIM.

### Strengths, Limitations, and Unanticipated Problems

The study’s strengths are the focus on both effectiveness and implementation through the type-1 hybrid approach; multilevel stakeholder engagement throughout the trial; a novel integration of RE-AIM, PRISM, and health behavior frameworks; the enhancement of a promising fully automated intervention; the scalability of this mobile or web-based intervention; the cost analysis; the longitudinal study design; and the inclusion of clinical outcomes. There are few interventions that have been evaluated to improve SSE among survivors of melanoma. No published intervention is fully automated, which represents a potentially cost-effective and scalable intervention delivery method. This study will advance the science of cancer survivorship by optimizing a promising intervention for an underserved group of survivors and preparing for widespread dissemination and implementation. Few studies have focused on implementation science aspects of consumer-facing digital interventions. Our use of a type-1 hybrid effectiveness-implementation trial will inform not only our understanding of the effects and implementation of MSS but also our understanding of future large-scale dissemination of self-administered web-based interventions. By evaluating and modeling the clinical outcomes, cost, and cost-effectiveness of MSS, our work will provide information about the costs associated with possible implementation and value.

There are at least 2 limitations. First, some individuals will not use MSS despite its high dissemination. Second, SSE outcomes are measured through self-report. However, this weakness is mitigated by the fact that self-report measures have excellent reliability and validity [14,66,95-100] and self-report has been recommended as the most appropriate assessment approach for wide-scale skin cancer risk reduction research [101].

### Future Directions

If effective, MSS could be disseminated and delivered via dermatologist practices, public health organizations such as the American Cancer Society, and nonprofit organizations focused on melanoma or in partnership with existing social media channels. Future research should evaluate potential dissemination and implementation strategies to reach survivors building upon what we will learn in this study about the contextual factors that may impede or promote the future reach, adoption, implementation, and maintenance of MSS.

## Appendix

Multimedia Appendix 1 Peer review report from the Science of Implementation in Health and Healthcare Study Section Healthcare Delivery and Methodologies Integrated Review Group Center For Scientific Review (SIHH, USA).

## Abbreviations

AIM: Acceptability of Intervention Measure
BCT: behavior change technique
CINJ: Cancer Institute of New Jersey
COREQ: Consolidated Criteria for Reporting Qualitative Research
CRGC: Cancer Registry of Greater California
IRB: institutional review board
LYG: life year gained
MSS: mySmartSkin
NJSCR: New Jersey State Cancer Registry
PHM: preventive health model
PRISM: Practical, Robust Implementation and Sustainability Model
RCT: randomized controlled trial
RE-AIM: Reach, Effectiveness, Adoption, Implementation, and Maintenance
SSE: skin self-examination
UC: usual care

## Data Availability

The data sets generated and analyzed during this study are available from the corresponding author upon reasonable request*.*

## References

[1] Survival rates for melanoma skin cancer. American Cancer Society.

[2] Kao SY, Ekwueme DU, Holman DM, Rim SH, Thomas CC, Saraiya M (2023). Economic burden of skin cancer treatment in the USA: an analysis of the Medical Expenditure Panel Survey Data, 2012-2018. Cancer Causes Control.

[3] Francken AB, Accortt NA, Shaw HM, Colman MH, Wiener M, Soong SJ, Hoekstra HJ, Thompson JF (2008). Follow-up schedules after treatment for malignant melanoma. Br J Surg.

[4] Francken AB, Bastiaannet E, Hoekstra HJ (2005). Follow-up in patients with localised primary cutaneous melanoma. Lancet Oncol.

[5] Leiter U, Buettner PG, Eigentler TK, Bröcker EB, Voit C, Gollnick H, Marsch W, Wollina U, Meier F, Garbe C (2012). Hazard rates for recurrent and secondary cutaneous melanoma: an analysis of 33,384 patients in the German Central Malignant Melanoma Registry. J Am Acad Dermatol.

[6] Christianson DF, Anderson CM (2003). Close monitoring and lifetime follow-up is optimal for patients with a history of melanoma. Semin Oncol.

[7] NCCN clinical practice guidelines in oncology: melanoma; version 4.2011. National Comprehensive Cancer Network.

[8] Huang CL, Provost N, Marghoob AA, Kopf AW, Levin L, Bart RS (1998). Laboratory tests and imaging studies in patients with cutaneous malignant melanoma. J Am Acad Dermatol.

[9] Lifchez SD, Kelamis JA (2010). Melanoma: workup and surveillance. Clin Plast Surg.

[10] Lisy K, Lai-Kwon J, Ward A, Sandhu S, Kasparian NA, Winstanley J, Boyle F, Gyorki D, Lacey K, Bishop J, Jefford M (2020). Patient-reported outcomes in melanoma survivors at 1, 3 and 5 years post-diagnosis: a population-based cross-sectional study. Qual Life Res.

[11] Francken AB, Shaw HM, Accortt NA, Soong SJ, Hoekstra HJ, Thompson JF (2007). Detection of first relapse in cutaneous melanoma patients: implications for the formulation of evidence-based follow-up guidelines. Ann Surg Oncol.

[12] Wolff T, Tai E, Miller T (2009). Screening for skin cancer: an update of the evidence for the U.S. Preventive Services Task Force. Ann Intern Med.

[13] (2021). NCCN Guidelines® insights - melanoma: cutaneous, version 2.2021. National Comprehensive Cancer Network.

[14] Weinstock MA, Risica PM, Martin RA, Rakowski W, Smith KJ, Berwick M, Goldstein MG, Upegui D, Lasater T (2004). Reliability of assessment and circumstances of performance of thorough skin self-examination for the early detection of melanoma in the Check-It-Out Project. Prev Med.

[15] Moore Dalal K, Zhou Q, Panageas KS, Brady MS, Jaques DP, Coit DG (2008). Methods of detection of first recurrence in patients with stage I/II primary cutaneous melanoma after sentinel lymph node biopsy. Ann Surg Oncol.

[16] McPherson M, Elwood M, English DR, Baade PD, Youl PH, Aitken JF (2006). Presentation and detection of invasive melanoma in a high-risk population. J Am Acad Dermatol.

[17] Leiter U, Garbe C (2008). Epidemiology of melanoma and nonmelanoma skin cancer--the role of sunlight. Adv Exp Med Biol.

[18] De Giorgi V, Grazzini M, Savarese I, Gori A, Papi F, D'Errico A, Scarfì F, Gandini S (2015). The impact of body area in melanoma self-detection: a retrospective study. Eur J Cancer Prev.

[19] Pollitt RA, Geller AC, Brooks DR, Johnson TM, Park ER, Swetter SM (2009). Efficacy of skin self-examination practices for early melanoma detection. Cancer Epidemiol Biomarkers Prev.

[20] Murali R, Haydu LE, Long GV, Quinn MJ, Saw RP, Shannon K, Spillane AJ, Stretch JR, Kefford RF, Thompson JF, Scolyer RA (2012). Clinical and pathologic factors associated with distant metastasis and survival in patients with thin primary cutaneous melanoma. Ann Surg Oncol.

[21] Berwick M, Armstrong BK, Ben-Porat L, Fine J, Kricker A, Eberle C, Barnhill R (2005). Sun exposure and mortality from melanoma. J Natl Cancer Inst.

[22] Schneider JS, Moore DH 2nd, Mendelsohn ML (2008). Screening program reduced melanoma mortality at the Lawrence Livermore National Laboratory, 1984 to 1996. J Am Acad Dermatol.

[23] Lo SN, Scolyer RA, Thompson JF (2018). Long-term survival of patients with thin (T1) cutaneous melanomas: a Breslow thickness cut point of 0.8 mm separates higher-risk and lower-risk tumors. Ann Surg Oncol.

[24] Robinson JK, Wayne JD, Martini MC, Hultgren BA, Mallett KA, Turrisi R (2016). Early detection of new melanomas by patients with melanoma and their partners using a structured skin self-examination skills training intervention: a randomized clinical trial. JAMA Dermatol.

[25] Gogebakan KC, Mukherjee K, Berry EG, Sonmez K, Leachman SA, Etzioni R (2021). Impact of novel systemic therapies on the first-year costs of care for melanoma among Medicare beneficiaries. Cancer.

[26] Serra-Arbeloa P, Rabines-Juárez ÁO, Álvarez-Ruiz MS, Guillén-Grima F (2017). Cost of cutaneous melanoma by tumor stage: a descriptive analysis. Actas Dermosifiliogr.

[27] Duffecy J, Sanford S, Wagner L, Begale M, Nawacki E, Mohr DC (2013). Project onward: an innovative e-health intervention for cancer survivors. Psychooncology.

[28] Regan MW, Reid CD, Griffiths RW, Briggs JC (1985). Malignant melanoma, evaluation of clinical follow up by questionnaire survey. Br J Plast Surg.

[29] Coups EJ, Manne SL, Stapleton JL, Tatum KL, Goydos JS (2016). Skin self-examination behaviors among individuals diagnosed with melanoma. Melanoma Res.

[30] Manne S, Lessin S (2006). Prevalence and correlates of sun protection and skin self-examination practices among cutaneous malignant melanoma survivors. J Behav Med.

[31] Loescher LJ, Harris RB, Lim KH, Su Y (2007). Thorough skin self-examination in patients with melanoma. Oncol Nurs Forum.

[32] Mujumdar UJ, Hay JL, Monroe-Hinds YC, Hummer AJ, Begg CB, Wilcox HB, Oliveria SA, Berwick M (2009). Sun protection and skin self-examination in melanoma survivors. Psychooncology.

[33] Manne SL, Heckman CJ, Kashy D, Lozada C, Gallo J, Ritterband L, Coups EJ (2020). Prevalence and correlates of skin self-examination practices among cutaneous malignant melanoma survivors. Prev Med Rep.

[34] Bowen DJ, Burke W, Hay JL, Meischke H, Harris JN (2015). Effects of web-based intervention on risk reduction behaviors in melanoma survivors. J Cancer Surviv.

[35] Loescher LJ, Hibler E, Hiscox H, Quale L, Harris R (2010). An internet-delivered video intervention for skin self-examination by patients with melanoma. Arch Dermatol.

[36] Robinson JK, Gaber R, Hultgren B, Eilers S, Blatt H, Stapleton J, Mallett K, Turrisi R, Duffecy J, Begale M, Martini M, Bilimoria K, Wayne J (2014). Skin self-examination education for early detection of melanoma: a randomized controlled trial of internet, workbook, and in-person interventions. J Med Internet Res.

[37] Robinson JK, Turrisi R, Stapleton J (2007). Efficacy of a partner assistance intervention designed to increase skin self-examination performance. Arch Dermatol.

[38] Coups EJ, Manne SL, Ohman Strickland P, Hilgart M, Goydos JS, Heckman CJ, Chamorro P, Rao BK, Davis M, Smith FO, Thorndike FP, Ritterband LM (2019). Randomized controlled trial of the mySmartSkin web-based intervention to promote skin self-examination and sun protection behaviors among individuals diagnosed with melanoma: study design and baseline characteristics. Contemp Clin Trials.

[39] Manne SL, Heckman CJ, Kashy DA, Ritterband LM, Thorndike FP, Lozada C, Coups EJ (2021). Randomized controlled trial of the mySmartSkin web-based intervention to promote skin self-examination and sun protection among individuals diagnosed with melanoma. Transl Behav Med.

[40] Mayer D, Layman A, Carlson J (2012). Sun-protection behaviors of melanoma survivors. J Am Acad Dermatol.

[41] Freiman A, Yu J, Loutfi A, Wang B (2004). Impact of melanoma diagnosis on sun-awareness and protection: efficacy of education campaigns in a high-risk population. J Cutan Med Surg.

[42] Bowen D, Jabson J, Haddock N, Hay J, Edwards K (2012). Skin care behaviors among melanoma survivors. Psychooncology.

[43] Oliveria SA, Christos PJ, Halpern AC, Fine JA, Barnhill RL, Berwick M (1999). Evaluation of factors associated with skin self-examination. Cancer Epidemiol Biomarkers Prev.

[44] Myers RE, Ross E, Jepson C, Wolf T, Balshem A, Millner L, Leventhal H (1994). Modeling adherence to colorectal cancer screening. Prev Med.

[45] Bruce AF, Theeke L, Mallow J (2017). A state of the science on influential factors related to sun protective behaviors to prevent skin cancer in adults. Int J Nurs Sci.

[46] Manne S, Jacobsen PB, Ming ME, Winkel G, Dessureault S, Lessin SR (2010). Tailored versus generic interventions for skin cancer risk reduction for family members of melanoma patients. Health Psychol.

[47] Manne SL, Coups EJ, Jacobsen PB, Ming M, Heckman CJ, Lessin S (2011). Sun protection and sunbathing practices among at-risk family members of patients with melanoma. BMC Public Health.

[48] Myers RE, Hyslop T, Wolf TA, Burgh D, Kunkel EJ, Oyesanmi OA, Chodak GJ (2000). African-American men and intention to adhere to recommended follow-up for an abnormal prostate cancer early detection examination result. Urology.

[49] Proctor EK, Colditz GA, Brownson RC (2012). Dissemination and Implementation Research in Health: Translating Science to Practice.

[50] Glasgow RE, Vogt TM, Boles SM (1999). Evaluating the public health impact of health promotion interventions: the RE-AIM framework. Am J Public Health.

[51] Blackman KC, Zoellner J, Berrey LM, Alexander R, Fanning J, Hill JL, Estabrooks PA (2013). Assessing the internal and external validity of mobile health physical activity promotion interventions: a systematic literature review using the RE-AIM framework. J Med Internet Res.

[52] Carnevale TD (2013). Universal adolescent depression prevention programs: a review. J Sch Nurs.

[53] Glasgow RE, Klesges LM, Dzewaltowski DA, Estabrooks PA, Vogt TM (2006). Evaluating the impact of health promotion programs: using the RE-AIM framework to form summary measures for decision making involving complex issues. Health Educ Res.

[54] Kessler RS, Purcell EP, Glasgow RE, Klesges LM, Benkeser RM, Peek CJ (2013). What does it mean to "employ" the RE-AIM model?. Eval Health Prof.

[55] Klesges LM, Dzewaltowski DA, Glasgow RE (2008). Review of external validity reporting in childhood obesity prevention research. Am J Prev Med.

[56] Kwan BM, Brownson RC, Glasgow RE, Morrato EH, Luke DA (2022). Designing for dissemination and sustainability to promote equitable impacts on health. Annu Rev Public Health.

[57] Michie S, Richardson M, Johnston M, Abraham C, Francis J, Hardeman W, Eccles MP, Cane J, Wood CE (2013). The behavior change technique taxonomy (v1) of 93 hierarchically clustered techniques: building an international consensus for the reporting of behavior change interventions. Ann Behav Med.

[58] Atkinson NL, Massett HA, Mylks C, Hanna B, Deering MJ, Hesse BW (2007). User-centered research on breast cancer patient needs and preferences of an internet-based clinical trial matching system. J Med Internet Res.

[59] Auxier B, Anderson M (2021). Social media use in 2021. Pew Research Center.

[60] Manne S, Buller D, Devine K, Heckman C, Pagoto S, Frederick S, Mitarotondo A (2020). Sun safe partners online: pilot randomized controlled clinical trial. J Med Internet Res.

[61] Heckman CJ, Riley M, Khavjou O, Ohman-Strickland P, Manne SL, Yaroch AL, Bhurosy T, Coups EJ, Glanz K (2021). Cost, reach, and representativeness of recruitment efforts for an online skin cancer risk reduction intervention trial for young adults. Transl Behav Med.

[62] Manne S, Day A, Coups EJ, Kashy D (2018). Sun safe partners: a pilot and feasibility trial of a couple-focused intervention to improve sun protection practices. Prev Med Rep.

[63] Manne S, Hudson S, Frederick S, Mitarotondo A, Baredes S, Kalyoussef E, Ohman-Strickland P, Kashy DA (2020). e-Health self-management intervention for oral and oropharyngeal cancer survivors: design and single-arm pilot study of empowered survivor. Head Neck.

[64] Melanoma warning signs. The Skin Cancer Foundation.

[65] Self-exams save lives: how to do a skin check. The Skin Cancer Foundation.

[66] Glanz K, Yaroch AL, Dancel M, Saraiya M, Crane LA, Buller DB, Manne S, O'Riordan DL, Heckman CJ, Hay J, Robinson JK (2008). Measures of sun exposure and sun protection practices for behavioral and epidemiologic research. Arch Dermatol.

[67] Lang KM, Little TD (2018). Principled missing data treatments. Prev Sci.

[68] Cho BH, Hicks KA, Honeycutt AA, Hupert N, Khavjou O, Messonnier M, Washington ML (2011). A tool for the economic analysis of mass prophylaxis operations with an application to H1N1 influenza vaccination clinics. J Public Health Manag Pract.

[69] Honeycutt AA, Khavjou OA, Bradley C, Neuwahl S, Hoerger TJ, Bellard D, Cash AJ (2016). Intervention costs from communities putting prevention to work. Prev Chronic Dis.

[70] Honeycutt AA, Khavjou OA, Jones DJ, Cuellar J, Forehand RL (2015). Helping the noncompliant child: an assessment of program costs and cost-effectiveness. J Child Fam Stud.

[71] Khavjou OA, Honeycutt AA, Hoerger TJ, Trogdon JG, Cash AJ (2014). Collecting costs of community prevention programs: communities putting prevention to work initiative. Am J Prev Med.

[72] Khavjou OA, Honeycutt AA, Yarnoff B, Bradley C, Soler R, Orenstein D (2018). Costs of community-based interventions from the Community Transformation Grants. Prev Med.

[73] Yarnoff B, Khavjou O, Bradley C, Leis J, Filene J, Honeycutt A, Herzfeldt-Kamprath R, Peplinski K (2019). Standardized cost estimates for home visiting: pilot study of the home visiting budget assistance tool (HV-BAT). Matern Child Health J.

[74] Kakushadze Z, Raghubanshi R, Yu W (2017). Estimating cost savings from early cancer diagnosis. Data.

[75] Guy JG Jr, Zhang Y, Ekwueme DU, Rim SH, Watson M (2017). The potential impact of reducing indoor tanning on melanoma prevention and treatment costs in the United States: an economic analysis. J Am Acad Dermatol.

[76] Guy GP Jr, Machlin SR, Ekwueme DU, Yabroff KR (2015). Prevalence and costs of skin cancer treatment in the U.S., 2002-2006 and 2007-2011. Am J Prev Med.

[77] Styperek A, Kimball AB (2012). Malignant melanoma: the implications of cost for stakeholder innovation. Am J Pharm Benefits.

[78] Alexandrescu DT (2009). Melanoma costs: a dynamic model comparing estimated overall costs of various clinical stages. Dermatol Online J.

[79] SEER cancer statistics review (CSR). National Cancer Institute.

[80] Haddix AC, Teutsch SM, Corso PS (1996). Prevention Effectiveness: A Guide to Decision Analysis and Economic Evaluation.

[81] Neumann PJ, Sanders GD, Russell LB, Siegel JE, Ganiats TG (2016). Cost-Effectiveness in Health and Medicine.

[82] Arias E, Bastian B, Xu J, Tejada-Vera B (2021). U.S. State life tables, 2018. National Vital Statistics Reports.

[83] Sampogna F, Paradisi A, Iemboli ML, Ricci F, Sonego G, Abeni D (2019). Comparison of quality of life between melanoma and non-melanoma skin cancer patients. Eur J Dermatol.

[84] Blackford S, Roberts D, Salek MS, Finlay A (1996). Basal cell carcinomas cause little handicap. Qual Life Res.

[85] Schlesinger-Raab A, Schubert-Fritschle G, Hein R, Stolz W, Volkenandt M, Hölzel D, Engel J (2010). Quality of life in localised malignant melanoma. Ann Oncol.

[86] Rhee JS, Matthews BA, Neuburg M, Smith TL, Burzynski M, Nattinger AB (2004). Skin cancer and quality of life. Dermatol Surg.

[87] Sampogna F, Tabolli S, Abeni D (2013). Impact of different skin conditions on quality of life. G Ital Dermatol Venereol.

[88] Schubert-Fritschle G, Schlesinger-Raab A, Hein R, Stolz W, Volkenandt M, Hölzel D, Engel J (2013). Quality of life and comorbidity in localized malignant melanoma: results of a German population-based cohort study. Int J Dermatol.

[89] Weiner BJ, Lewis CC, Stanick C, Powell BJ, Dorsey CN, Clary AS, Boynton MH, Halko H (2017). Psychometric assessment of three newly developed implementation outcome measures. Implement Sci.

[90] De Marchis EH, Hessler D, Fichtenberg C, Adler N, Byhoff E, Cohen AJ, Doran KM, Ettinger de Cuba S, Fleegler EW, Lewis CC, Lindau ST, Tung EL, Huebschmann AG, Prather AA, Raven M, Gavin N, Jepson S, Johnson W, Ochoa E Jr, Olson AL, Sandel M, Sheward RS, Gottlieb LM (2019). Part I: a quantitative study of social risk screening acceptability in patients and caregivers. Am J Prev Med.

[91] Proctor E, Silmere H, Raghavan R, Hovmand P, Aarons G, Bunger A, Griffey R, Hensley M (2011). Outcomes for implementation research: conceptual distinctions, measurement challenges, and research agenda. Adm Policy Ment Health.

[92] Hsieh HF, Shannon SE (2005). Three approaches to qualitative content analysis. Qual Health Res.

[93] Fielding NG, Fielding JL (1986). Linking Data.

[94] Creswell JW, Clark VL (2007). Designing and Conducting Mixed Methods Research.

[95] Weinstock MA, Martin RA, Risica PM, Berwick M, Lasater T, Rakowski W, Goldstein MG, Dubé CE (1999). Thorough skin examination for the early detection of melanoma. Am J Prev Med.

[96] Glanz K, Schoenfeld ER, Steffen A (2010). A randomized trial of tailored skin cancer prevention messages for adults: project SCAPE. Am J Public Health.

[97] O'Riordan DL, Glanz K, Gies P, Elliott T (2008). A pilot study of the validity of self-reported ultraviolet radiation exposure and sun protection practices among lifeguards, parents and children. Photochem Photobiol.

[98] O'Riordan DL, Nehl E, Gies P, Bundy L, Burgess K, Davis E, Glanz K (2009). Validity of covering-up sun-protection habits: association of observations and self-report. J Am Acad Dermatol.

[99] Hillhouse J, Turrisi R, Jaccard J, Robinson J (2012). Accuracy of self-reported sun exposure and sun protection behavior. Prev Sci.

[100] Køster B, Søndergaard J, Nielsen JB, Olsen A, Bentzen J (2018). Reliability and consistency of a validated sun exposure questionnaire in a population-based Danish sample. Prev Med Rep.

[101] Glanz K, Mayer JA (2005). Reducing ultraviolet radiation exposure to prevent skin cancer methodology and measurement. Am J Prev Med.

