# Supplementary material for: A Digital Intervention to Improve Skin Self-Examination Among Survivors of Melanoma: Protocol for a Type-1 Hybrid Effectiveness-Implementation Randomized Trial
Source: JMIR Res Protoc. 2024 Feb 12;13:e52689. doi: 10.2196/52689 (PMC10897801; doi:10.2196/52689)
Supplement: Multimedia Appendix 1 [file resprot_v13i1e52689_app1.docx]

**Summary of Comments**

| **Skin Self-Check Module Feedback** | | |
| --- | --- | --- |
| **Feedback** | **Solution** | **Team Decision** |
| User did not realize they can scroll | Condense as many screens as possible so that the content can fit on screen without the need to scroll  Add a scroll bar on the side so user knows they can scroll | Implemented |
| User does not know how to get back to the full body map when they are in a body part section | Get rid of the back arrow on this page to avoid confusion  Change “More” button to say “Body Map” so user knows that button will take them back to the main screen | Implemented |
| User tries to click on the body part graphics rather than the text below the graphics | Make each body part graphic clickable | Implemented |
| User found it confusing to log spots on back because there are zones and regions | Not implementing because need to have different regions for user to keep track of the spot location | Not implemented |
| Nothing in body map that tracks progress, progress tracker would also serve as motivation for user | Add in a body part number tracker to track progress through exam | Implemented |
| Add a zoom option throughout the app | Implement the ability to zoom | Implemented |
| Some of the text is hard to read | Bold and increase size of text | Implemented |
| Add tutorials throughout the app for further directions and instructions | Add an information button on top of any confusing screens | Implemented |
| Clarify characteristics page within the body map | Condense letter boxes, give clear directions, and add ABCDEF guide for reference in case user forgets what each letter stands for | Implemented |
| Having a diameter is confusing as a characteristic | Not implementing because this is an important characteristic for SSE  Clarify directions to make less confusing for user | Not implemented |
| **Goal Setting Module Feedback** | | |
| More thorough directions and instructions throughout app | Clarified instructions, add tutorials throughout | Implemented |
| Don’t need two summary screens for goal setting | Combine two summary screens into one, removing unnecessary information  Utilize the formatting and layout of the second screen | Implemented |
| Broad goal screen isn’t clear enough without interviewer instruction | Work on making this screen clearer using further details into which each goal means based on user’s current habits | Implemented |
| If user wants to set another goal after reaching the summary screen, they are not sure how to do that | Provide a link in the summary screen that goes back to the first page if user wants to set another goal | Implemented |
| **General Feedback** | | |
| Use the MSS app to send notifications and reminders related to other types of checks (breast, pap) |  | Undecided |
| Have an option to communicate with other users to boost motivation and sense of community | Cannot implement because would be considering sharing PHI | Not implemented |
| Coaching options for support | State that research team is available for questions | Implemented |
| Ability to connect with dependents or support person through the app | User would need to provide support person’s information | Undecided |
| Notifications through app; SSE reminders | The MSS app will be programmed to remind users to complete their SSE | Implemented |
| More user control of frequency of reminders | Option to opt out or change frequency of reminders | Implemented |
| Message/notification portal in app | Will not be sending out enough notifications that a message portal is necessary | Not Implemented |
| Use phone calls only if user is inactive for a long period of time or expresses the need for help | Communicate to user via app notifications, text, and email; only call participants if they have been inactive or requests a call from the study team | Implemented |
| Change the name of “prizes” to “tools” so that it doesn’t seem as much like a game but rather helpful items they can use to help with SSE and sun safety |  | Implemented |
| Homepage has too much going on | Remove information about goals and myStuff on homepage | Implemented |
| Display of progress on homepage is not clear | Make it look more like a bar graph - simpler and clearer | Implemented |
| Include time indication for each chapter so user knows how long it will take | Add estimated minutes to complete next to each chapter | Implemented |
| Instructions throughout app are not clear enough | Use clearer, simpler language; added tutorials for visual aides | Implemented |
| Simplify functionality; some components of the app are too complex | Include tutorials throughout the application; simplified functionality when possible | Implemented |
| User wants more visuals included throughout the app | Add more graphics and guides for visual aid | Implemented |
| Mixed feedback on the gamification components of the app | Reduce and simplify the gamification so even the users that don’t enjoy will be unbothered by game-aspects | Implemented |
| Make content accessible via phone, computer, tablet, etc. | The mobile version will be accessible via phone and tablet with separate version tailored for computer access | Implemented |

Note: MSS: mySmartSkin; SSE: Skin self-exam; PHI: Protected health information
